# Book Reviews

**Published:** 1910-06

**Authors:** 


### BOOK REVIEWS.

EXAMINATION OF THE URINE: A MANUAL FOR STUDENTS and Practitioners. By G. A. DeSantos Saxe, M. D., Instructor in Genito-Urinary Surgery, New York Post-Graduate Medical School and Hospital. Second edition, enlarged and reset. 12 mo. of 448 pages, illustrated. Philadelphia and London. W. B. Saunders Company, 1909. Cloth, $1.75 net.

This useful little volume on urinalysis has so impressed the author with its merit that he wishes to recommend it to his students as a text-book for the coming session. It may well be considered a safe, concise and valuable guide for both the general practitioner in his work and for the student in his laboratory course. Special attention has been paid to technics and the interpretation of findings.

---

PULMONARY TUBERCULOSIS AND ITS COMPLICATIONS. By Sheman G. Bonney, M. D., Professor of Medicine, Denver and Gross College of Medicine, Denver. Octavo of 955 pages, with 243 original illustrations, including 31 in colors and 73 X-ray photographs. Philadelphia and London: W. B. Saunders Company, 1910. Cloth, $7.00 net; half morocco, $8.50 net.

This splendid monograph following the first edition so closely affords ample proof of its value both by the cordial reception of the work and by a careful perusal of its pages. The importance of the subject and the need of more than the ordinary text book knowledge of tuberculosis is recognized by all who fight this treacherous plague. Every phase of the subject is exhaustively discussed in a clear, impressive manner by Bonney. The entire work has been revised with extreme care and much new subject material added. The valuable contributions made at the recent International Congress on Tuberculosis held in Washington have been carefully reviewed. Five new chapters have been added and 51 additional illustrations. The book cannot be too highly commended.

